# Uncovering Sleep Behaviour in Women’s Football: What Evidence Do We Have?

**DOI:** 10.1007/s40279-025-02247-w

**Published:** 2025-08-25

**Authors:** Shona L. Halson, Amy Bender, Glyn Howatson, Charles Pedlar

**Affiliations:** 1 School of Behavioural and Health Sciences, Australian Catholic University, 1100 Nudgee Road, Brisbane Campus McAuley, Banyo, QLD Australia; 2 Sports Performance, Recovery, Injury and New Technologies (SPRINT) Research Centre, Australian Catholic University, Brisbane, QLD Australia; 3 Faculty of Kinesiology, University of Calgary, Calgary, AB Canada; 4 Faculty of Health and Life Sciences, Northumbria University, Newcastle upon Tyne, UK; 5 Water Research Group, School of Biological Sciences, North-West University, Potchefstroom, South Africa; 6 Faculty of Sport, Technology and Health Sciences, St Mary’s University, Twickenham, London, UK; 7 Institute of Sport, Exercise and Health, Division of Surgery and Interventional Science, University College London, London, UK; 8 Orreco Ltd., Business Innovation Centre, University of Galway, Galway, Ireland

## Abstract

Sleep plays an important role in performance, health and well-being, yet may represent a challenge to many female football players. Areas of the brain that are involved in the regulation of sleep contain receptors for the ovarian hormones, estrogen and progesterone. While limited evidence exists describing sleep across the menstrual cycle in elite female football players, related data suggest that female athletes may report poor subjective sleep, despite appropriate objectively measured sleep quality and quantity, particularly prior to or during menstruation. Some of the precipitators of poor sleep in female athletes may include: travel and jetlag, caffeine consumption, light exposure, competing at night, menstrual cycle symptoms, menstrual cycle dysfunction, low iron status and performing caring responsibilities. This article discusses potential approaches to protect, assess and provide interventions to support sleep in female football players. Despite the evidence base of research being predominantly studies of male athletes, there are a number of specific recommendations that can be made for female athletes. These include advice regarding methods to assess sleep and provide interventions based on resource availability, monitoring and managing menstrual cycle symptoms and menstrual dysfunction, and consideration of mitigating strategies to reduce the effects on known sleep disruptors. Many female footballers navigate unique challenges related to sleep; however, with appropriate support from coaches and sport science and sports medicine practitioners, an appropriate support network can be provided to not only optimise performance, but the physical and mental health of female athletes.

## Key Points

**Table:** 

Female athletes obtain sufficient sleep when measured objectively; however, when subjectively rating sleep, female athletes often report sleep complaints. Subjective reports of poor sleep are more likely to be evident during or immediately prior to menstruation, potentially due to an association between sleep and menstrual cycle symptoms.
Reasons for poor sleep in female athletes may include: travel and jetlag, caffeine consumption, light exposure, competing at night, menstrual cycle symptoms, menstrual cycle dysfunction, low iron status and caring responsibilities.
Despite known disparities in resource availability, there are a number of effective methods to both assess sleep and provide interventions, which range from questionnaires and online education through to polysomnography and expert consultation.

## Background

Sleep is an essential physiological process for an athlete to recover from the physical and mental demands of sport. Optimal sleep plays a role in how athletes perform and may also influence health and well-being. Much of what is known about the importance of sleep in sport is based on research in men. For example, with more sleep, male basketball players improved athletic performance of free throws by 11%, three-point shots of 14%, sprint times by 4% and reaction time by 12% [1]. Other reasons why sleep is important for an athlete are related to reductions in stress hormones in male rugby players [2], increases in testosterone levels with sleep extension in young men [3], and a decreased risk for illness [4] and injury [5, 6] in mixed male/female cohorts.

To provide comprehensive information for this review, we conducted a literature search using PubMed and Web of Science with combinations of the following search terms: ‘female’, ‘football’, ‘team sport’, ‘sleep’, ‘wearables’, ‘iron’, ‘nutrition’ and ‘monitoring’. We screened the search results to identify publications relevant to sleep in female football players.

### Football-Specific Challenges

Participation and fandom around women’s football has grown significantly in recent years, which is strongly supported by the Fédération Internationale de Football Association’s (FIFA’s) strategic ambition to grow global female player participation to 60 million by 2026 [7]. In the 2023 FIFA Women’s World Cup, an unprecedented level of resources were provided to the participating nations and demonstrated what is possible for the women’s game. It culminated in a 32-team, 64-match competition that sold over 1.95 million stadium tickets and generated $570 million in revenue, in a tournament that attracted global attention with > 3 billion content views [8]. Despite the growth of female football, there remains a huge disparity within the game between male and female individuals. When coupled with the added inequity of resources available between female footballing nations, there is the potential for increased inequality, particularly for financially challenged nations and those athletes from areas where participation in football can be challenging because of cultural backgrounds.

Despite the inequality of resources between teams from grassroots to the elite international level, many challenges to the sleep–wake cycle remain similar regardless of geography or demographics. At the elite level, long haul travel is often frequent and crosses multiple time zones, which can result in jetlag and travel fatigue. Match schedules may result in finish times late in the evening, causing substantial disturbances to sleep patterns. Finally, caffeine has been reported to be used by ~ 23% of an elite female football cohort [9]. Given that caffeine ingestion can disturb sleep when consumed a few hours before planned sleep [10], there is the potential for further sleep disturbances for those caffeine consumers competing in evening fixtures.

Sleep is undoubtedly an important component of athletic performance, but there are many challenges to overcome in football that require consideration. By understanding the environment female players are exposed to, in addition to specific attention to an individual’s needs, there is an opportunity to implement strategies to support sleep and thereby reduce fatigue and improve performance.

## Sleep in Female Athletes

Female athletes are reported to obtain sufficient sleep when measured objectively (e.g. using an activity monitor); however, when subjectively rating sleep (e.g. diary or questionnaire), female athletes often report sleep complaints [11]. The exact reason for this discrepancy is unknown, although it is well established that the agreement between objective and subjective assessments of sleep is relatively weak and should not be considered as proxy measures of each other [12]. This may be because of the differences in methodology for objectively assessing sleep (polysomnography vs wearable devices) and differences in the assessment of subjective sleep quality (diaries vs questionnaires). It could also be related to a lack of sensitivity of an objective measure to capture changes in subjective sleep quality. For example, female individuals have higher rates of depression and anxiety [12], which is more associated with poorer subjective sleep quality versus a more subtle impact on objective sleep that may not be captured with actigraphy or other consumer wearable devices.

A systematic review with meta-analysis investigating sleep in female athletes found high variability in objectively measured sleep between studies as well as impaired sleep following training as well as before and after competition [11]. Therefore, variability in sleep in female athletes is likely high between individuals and potentially within individuals, depending on the nature of training and competition.

When compared with male athletes, while appreciating variability in existing findings, data suggest that female athletes have longer sleep durations than male athletes [13]. While no difference in objectively measured sleep duration was observed between sexes in 175 athletes (only 17% female) across 12 sports, habitual sleep onset times were earlier in female athletes [14]. Importantly, in this and other studies, the comparison was not sport specific and female and male athletes were not compared within the same sport because of low participant numbers. As habitual sleep duration differs as a function of sport, sex differences may be obscured by the type of sport the athletes participate in [14]. Female athletes have also been shown to have better sleep quality than male athletes when measured objectively [15] and have similar or slightly lower sleep quality when measured subjectively [13], including longer sleep onset latencies [16]. This may influence the higher daytime dysfunction that has been reported in elite female athletes when compared with male athletes [16]. It is evident from the lack of research investigating sleep in female athletes and sleep across the menstrual cycle that additional longitudinal monitoring in elite female athletes is required.

Sleep duration has been shown to be reduced in female athletes following evening matches during an international soccer tournament [17]. While a number of players had reduced sleep duration on any given day of the tournament, the largest number of players receiving less than 7 h of sleep was evident after evening matches. This was largely a consequence of later sleep-onset times and reinforces advice to aim to protect sleep duration by allowing players to extend their sleep in the morning. In this study, sleep quality was unaffected by training or matches [17].

### Sleep and Ovarian Hormones

The biological processes that occur during sleep might be influenced by natural changes in endogenous hormone levels across a menstrual cycle and exogenous hormones from hormonal contraception [18–20]. Receptors for estrogen and progesterone are found in the central nervous system in areas of the brain that are also involved in sleep regulation [21]. Therefore, there is potential that sleep characteristics may vary at different timepoints during the menstrual cycle and that some of the variability in sleep observed in previous research might be due to natural changes in ovarian hormone levels, which to date has not been considered in previous sleep research in female athletes.

Available data in non-athletes suggests that sleep is relatively stable across the menstrual cycle when measured objectively. In one of the most comprehensive studies to date, Driver et al. [22] monitored nine healthy female participants using the gold standard of sleep measurement, polysomnography, every second night for the duration of the participants’ menstrual cycle. No changes in sleep metrics were observed, with the exception of increased sleep spindle activity in the luteal phase [22]. Similar findings have also been reported in other studies [23–25], including those using wearables [26].

Two studies, however, have reported changes in objective measures of sleep at different timepoints of the menstrual cycle, including less deep sleep during menses compared with non-menses days in collegiate athletes [27] and shorter rapid-eye movement latency during the luteal versus follicular phase (although no other sleep changes were observed) in healthy women (non-athletes) [28]. However, because of methodological differences in the assessment of menstrual cycle phases, sleep monitoring techniques and study duration, there is a lack of definitive evidence of changes in objective measures of sleep across the menstrual cycle. However, as observed above in female athletes, subjective measures of sleep might differ across the menstrual cycle.

Poor subjective sleep quality has been identified during phase one (early follicular) of the menstrual cycle and prior to menstruation in athletes and non-athletes [29–31], with the suggestion that ovarian hormone levels may be related to subjective sleep reports [31]. Self-reported sleep quality was decreased during menstruation and pre-menstruation compared with other phases of the menstrual cycle in young healthy non-athletes [32]. However, there were no differences in other self-reported sleep measurements, such as total sleep time, sleep onset latency, and the number and duration of awakenings, during any phase of the menstrual cycle [32]. From the available literature, it appears that subjective sleep measures are likely to be affected more than objective sleep measures; furthermore, subjective reports of sleep are more likely to be evident during or immediately prior to menstruation.

### Sleep and Hormonal Contraception

It has been reported that 28% of female football players in the Women’s Super League competition use hormonal contraception [33]. This figure is similar to that reported in the UK and US general population [33], but lower than the approximately 50% reported in other elite athletes [33–35]. The influence of hormonal contraception on sleep has been investigated in a small number of studies in non-athletes. Hachul et al. [36] found that most (66%) hormonal contraception users had poor ratings of sleep quality compared with non-hormonal contraception users. Reduced sleep duration and a high insomnia severity index have also been found in female individuals taking third-generation hormonal contraception (i.e. progestin derived from levonorgestrel) in comparison to non-hormonal contraception users [37]. However, increased sleep duration has also been reported in progesterone-only contraception users [38]. Importantly, sleep has not been objectively measured in any of the above studies. A recent review on the effects of hormonal contraceptives on the sleep of female individuals of reproductive age [39] reported that contraceptive use could impact sleep, yet the direction of this relationship is not clear. Increased sleepiness, insomnia symptoms, decreased sleep efficiency and a reduced overall sleep quality are the most common subjective findings, with hormonal intrauterine contraceptives associated with less negative effects on sleep.

### Menstrual Cycle Symptoms and Sleep

In a population of active female individuals with naturally cycling sex hormones, changes in mood and anxiety (90.6%), tiredness and fatigue (86.2%), stomach cramps (84.2%) and breast pain (83.1%) have been reported during the menstrual cycle [40]. In addition, athletes may be anxious, distracted or have fear associated with menses [41]. Symptoms have been reported to be more common during the late luteal phase and/or during menses [42], which aligns to the timepoint most associated with the poorer subjective sleep complaints mentioned above. A recent study investigating objectively measured sleep across a menstrual cycle in elite female footballers found an association between the total number of menstrual cycle symptoms reported and increased wake after sleep onset, indicating reduced sleep quality with a higher number of symptoms [43]. While there is limited evidence of an association between sleep and menstrual cycle symptoms, given the potential for symptoms to interfere with or influence sleep in a negative manner, assessing (and managing) symptoms alongside sleep could be important when monitoring elite athletes.

### Menstrual Dysfunction and Sleep

Menstrual dysfunction refers to a deviation from the regular cyclical pattern indicative of a eumenorrheic cycle, with types of menstrual dysfunctions classified as ovarian dysfunction, abnormal uterine bleeding and pain, and other symptomology [44]. Specific data regarding the prevalence of menstrual dysfunction in football players are limited; however, a recent review suggests that menstrual dysfunction is higher in athletes than the general population [45]. This review suggests that 20% of female football players experience menstrual cycle dysfunction [45]. While there is a lack of consistent findings regarding changes in sleep at various timepoints in the menstrual cycle and with hormonal contraception, changes in subjective sleep are evident in female individuals with menstrual dysfunction [25, 46–48]. For example, female individuals with severe pre-menstrual syndrome frequently report sleep disturbances during the late-luteal phase [49] and dysmenorrheic female individuals reported poorer subjective sleep quality during menstruation compared with other times during the menstrual cycle in comparison to healthy controls [46]. This is unsurprising given the bi-directional relationship between sleep and pain [50] and the reduction in nocturnal pain and improvement in the quality of sleep in women with primary dysmenorrhea following anti-inflammatory drug administration (diclofenac) [51].

Similar to research investigating sleep in eumenorrheic female individuals, findings of objective sleep measures are less consistent in female individuals with menstrual dysfunction. Despite significant subjective sleep complaints in female individuals with premenstrual syndrome and premenstrual dysphoric disorder, objectively measured sleep using polysomnography was unchanged [23, 25]. However, disturbed sleep (reduction in sleep efficiency, rapid eye movement sleep and increased stage one sleep) has been reported in participants with primary dysmenorrhea [23, 25].

## Sleep Disturbances

**Fig. 1:**
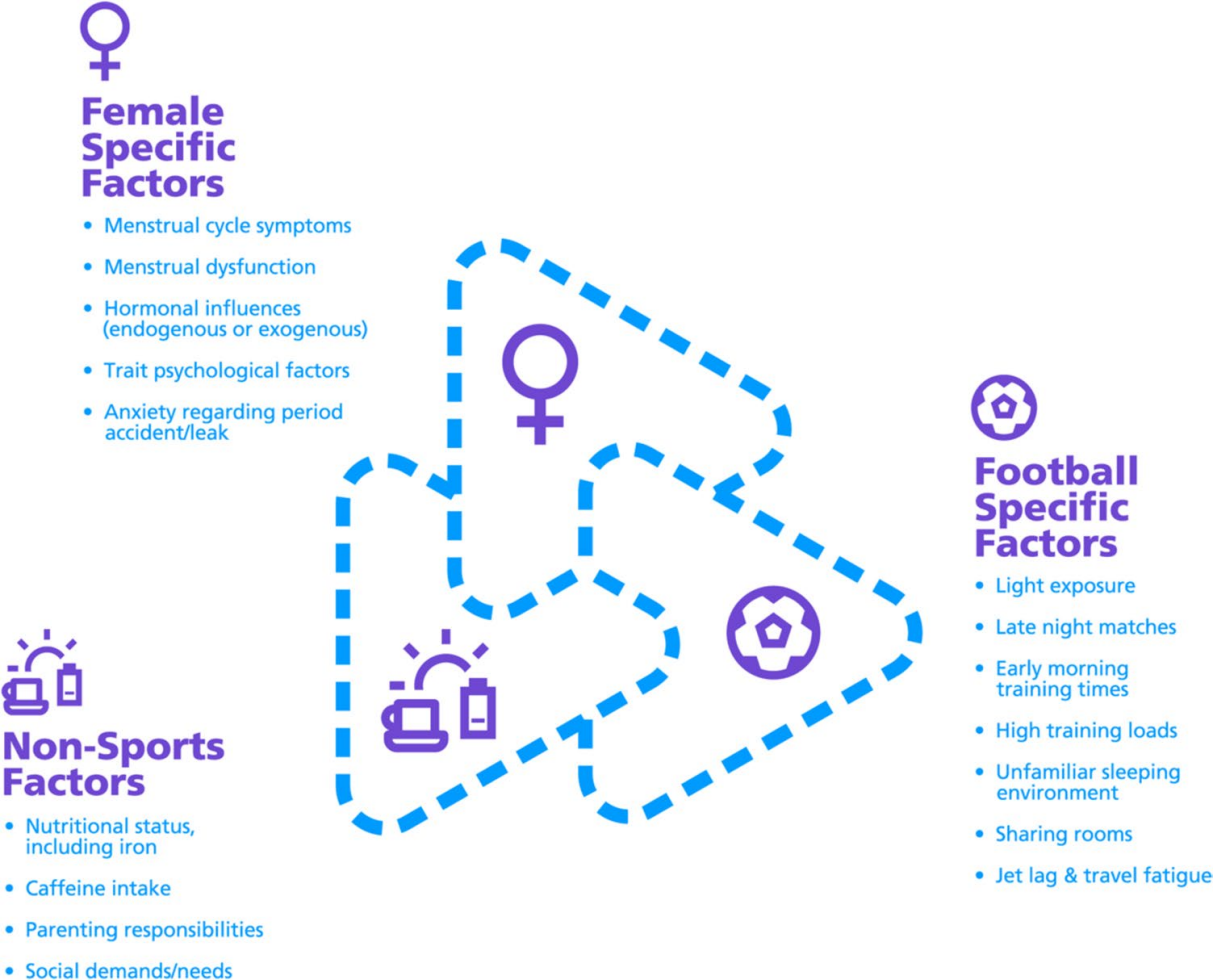
Potential factors contributing to sleep disturbance in athletes, including football, non-sport and female-specific factors

There are many factors that may contribute to sleep disturbance in athletes, including both non-sport factors and sport-specific factors (Fig. 1). This figure highlights some of the more common sleep disturbances experienced by athletes, with an additional focus on potential unique female factors that may be implicated in disturbed sleep.

### Light Exposure

Football presents specific sleep challenges including frequent late-night matches and travel across multiple time zones. Evening bright/blue light exposure, such as “being under the lights” for a match, has the potential to supress melatonin secretion and reduce sleep quality [55]. However, strategies such as wearing blue-light blocking glasses after competition may help reduce further exposure and promote melatonin, though the evidence remains mixed [56]. Furthermore, reducing bright light exposure in the home environment could also be a useful strategy. Research from Melbourne, Australia found that nearly half of homes assessed during Autumn–Winter had lighting bright enough to decrease melatonin production by 50% [57]. This issue could be more pronounced during the follicular phase of the menstrual cycle, when sensitivity to light is increased [58]. Therefore, it is important to consider strategies post-competition such as wearing blue-light blocking glasses and dimming lights before bedtime.

Another common source of disruptive light exposure comes from electronic devices, which can negatively affect both athletes and the general population. In a study involving elite youth athletes, many admitted to poor sleep habits, including engaging in stimulating activities such as using devices before bed [59]. Excessive social media use has been shown to contribute to poor sleep quality and a longer time to fall asleep [60]. Athletes frequently report using devices in the 30 min before sleep, often leading to difficulty falling asleep [61]. Reducing screen time and social media use before bed can be essential for improving both sleep onset and quality [59].

### Caffeine

Approximately 47% of football athletes consume caffeine with no differences in consumption between male and female individuals [62]. Consumption of caffeine by footballers is because of the widely accepted benefits of caffeine on performance and positive effects on aerobic endurance and passing accuracy in football players [63, 64]. Most of the caffeine research to date has been conducted in male athletes, and there is a paucity of similar research in female athletes. Interestingly, there is evidence of a difference in the cardiovascular response to caffeine between female and male individuals thought to be related to sex hormones. Heart rate decreased to a greater extent in male individuals than female individuals with caffeine administration [65], and greater (positive) effects on mood are observed in the follicular phase of the menstrual cycle compared with the luteal phase [66]. Furthermore, there is some evidence that caffeine use is associated with menstrual irregularities [67] and worse menstrual cycle symptoms [68], along with a greater elimination half-life (7.9 h vs 5.4 h) in those taking oral contraceptives containing estrogen [69]. Therefore, caffeine strategies for female players should be applied with caution and used strategically.

When examining the influence of caffeine on sleep in athletes, male triathletes took longer to fall asleep, had lower amounts of rapid eye movement sleep and lower sleep durations after consuming caffeine [70]. The later the timing of caffeine intake, the greater potential for sleep disturbances exists because of the time taken to metabolise caffeine (4–6 h half-life). For instance, 85% of male Super Rugby players had higher caffeine levels after an evening game compared with pre-game levels [71]. In this study, athletes, on average, went to bed 3 h later than usual, slept 1.5 h less, experienced a longer time to fall asleep and had reduced sleep efficiency. Notably, 20% of the athletes reported not sleeping at all after the game. Considering the time required to recover from such sleep deprivation, it could be argued that caffeine use might be counterproductive long term. Additionally, the impact of caffeine on performance may vary depending on rates of caffeine metabolism. For example, a dose of 4 mg per kg of body weight can enhance endurance cycling performance, but athletes with slower caffeine metabolism saw their performance decrease by 14% compared with a placebo in a study of male triathletes [72]. Furthermore, research on trained male runners found no significant improvement in 800-m sprint performance with caffeine compared with a placebo, but caffeine intake was linked to poorer sleep quality, more time awake and more frequent awakenings that night [73]. It is also important to note that all the studies referenced above involved only male athletes.

### Nutrient Intake and Sleep

The relationship between nutrition and sleep has gained significant interest in recent years, with evidence suggesting that macronutrients and/or dietary supplements such as carbohydrate, α-lactalbumin, tart cherry juice, valerian, L-theanine and nucleotides have the potential for enhancing sleep [52, 53]. While there is a lack of female athlete-specific nutrition and sleep research, a recent paper by Condo et al. [54] reported that objectively measured sleep duration and quality were associated with nutrient intake in elite female Australian Rules Football players. A higher carbohydrate intake was associated with reduced sleep quality, and a higher saturated fat intake associated with reduced sleep onset latency. Higher iron intake was associated with longer sleep duration and higher iron, zinc and vitamin B_12_ intakes were associated with higher sleep quality. However, higher vitamin E intake was associated with reduced sleep efficiency [54]. These data suggest that there might be a relationship between sleep and nutrient intake in elite female athletes. Although the potential mechanisms are not clear, micronutrient supplementation might improve sleep in athletes with dietary deficiencies, and careful consideration should be given to macronutrient intake to optimise the balance between training and performance, without negatively influencing sleep.

Iron deficiency has been reported to occur in 15–35% of female athletes, which may be a result of regular menstrual blood loss combined with inadequate iron intake, and/or the interaction between female sex hormones and iron metabolism [74]. In a study of elite young (aged 13–17 years) German female football players, 69% of players were below the recommended daily allowance for iron intake [75]. Iron plays a role in neurotransmitter synthesis in the brain and iron deficiency has been associated with a range of sleep disorders including restless legs syndrome and periodic limb movement disorder, which may not be detectable with actigraphy or other consumer wearable devices [76]. Given the potential for iron deficiency in female athletes and the possible link between iron deficiency and sleep disturbance, assessing iron status and supplementing when deficient may be important for optimising sleep.

### Travel

Both ground and air travel are common in female football players and managing performance while jet-lagged or fatigued because of travel is common. Jet lag occurs when internal circadian rhythms are desynchronised with the external environment after crossing several time zones. Jat lag differs from travel fatigue, which arises from the physical demands of travel, though both can impact athletes. Research on reducing travel fatigue in athletes is limited, but it is clear that recovery time is essential. Travelling across time zones requires a rapid adjustment of circadian rhythms to maintain peak performance, but re-synchronisation happens slowly, about 1 h per day for eastward travel and 2 h per day for westward travel [77]. These disruptions can significantly affect sleep, with westward travel causing difficulty in waking up too early and eastward travel making it hard to fall asleep. To help manage this, athletes should adjust their travel schedules to ease the circadian shift when possible. For example, early morning flights are ideal for eastward travel. On short trips, it is often best to remain on the clock time of the home time zone to minimise disruption and prevent jet lag on the return. For longer trips, shifting the body’s internal clock before departure and banking extra sleep can make the adjustment easier, although this is often not practical in athletes. Detailed guidelines on managing jet lag and travel fatigue in athletes are available in a recent review [77].

Adapting to a new time zone can be aided by paying attention to external cues that regulate the circadian system, such as light exposure and meal timing. When travelling west, it helps to avoid light in the morning and seek light in the evening to delay the body clock. Traveling east, however, requires morning light exposure while avoiding it in the evening to promote an advance in circadian rhythms. Because controlling natural light exposure can be challenging, artificial light may be useful. Adjusting meal times can also support the desired shift, particularly by helping to regulate peripheral clocks, such as the liver clock, though adjusting meal times has less influence on the brain’s master clock [78]. Changing meal schedules may also alleviate gastrointestinal issues, one of the key symptoms of jet lag.

### Sharing Rooms

For many players, sharing rooms when travelling for matches can impair sleep. Only one study to date has compared sharing rooms to sleeping in individual rooms and while the participants were football players, they were male athletes [79]. However, reduced objective and subjective sleep quality was reported for most players when sharing rooms versus when sleeping in individual rooms. This reduction in sleep quality might have been due to post-training or post-match social activities, and/or conscious attention towards the other team mate, including noise and space consideration [79]. While we have limited data on the effects of sharing rooms, it appears sharing rooms could decrease sleep quality, although the effect on sleep is likely variable between individuals. Therefore, considerations of the effects of sharing rooms on subsequent sleep should be made. For example, pairing of players according to chronotype (early or late chronotype), behaviours (i.e. social media/phone use), and familiarity and comfort with each other is important.

### Sleep Medication

Few data exist describing sleep medication use among female athletes. The Pittsburgh Sleep Quality Index (PSQI), a tool widely used to screen athletes sleep quality, includes a question about sleep medication use. Cameron et al. [80] reported regular sleep medication use in 1% (using sleep medication three or more times per week) and 3% (reporting one to two times per week) in a mixed cohort of American professional and amateur athletes but did not report specific data for female individuals. Halson et al. [16] reported no sleep medication use among a large predominantly female sample of Australian Olympic athletes. Two PSQI studies specifically in female soccer players did not specifically report the responses to the questions about medication use [81, 82].

Sleep medication use may provide an effective strategy for helping athletes with clinically diagnosed sleep problems; however, routinely using sleep medication carries the risk of habit formation and addiction [83]. Arguably the one exception is melatonin, which appears to carry a lower risk and may be an effective recovery aid [84]; however, all the studies to date in football have been conducted in male individuals [85]. Female football players should therefore consult with suitably qualified medical professionals before using sleep medication including melatonin.

### Caring Responsibilities

Female athletes face significant challenges balancing their sports careers with the responsibilities of parenthood, which can profoundly impact their sleep patterns. Women tend to experience more sleep disruptions because of increased night-time caregiving responsibilities. In the first 3 months after giving birth, mothers sleep 1 h less each night than pre-pregnancy sleep compared with a decrease of only 15 min per night in fathers [86]. These changes in sleep patterns improve as the child gets older but may take up to 6 years after birth for a mother’s sleep duration and quality to fully recover to levels prior to pregnancy [86].

Sleep deprivation can lead to compromised athletic performance, an increased risk of injuries and hindered recovery processes [87]. Moreover, the dual role of athlete and parent often requires female football players to manage extensive schedules, including training sessions, matches and childcare duties, which can further exacerbate their sleep quality and overall well-being. Thus, while excelling in their athletic endeavours, female football players navigate unique challenges related to childcare responsibilities that necessitate attention to help adequate recovery after giving birth, a strengthened support system, and special attention to sleep and recovery strategies.

With the expansion of women’s professional and semi-professional sports leagues, more opportunities have emerged for female athletes to compete at high levels. However, significant financial disparities persist between male and female athletes, creating challenges that often require elite female players, especially in sports like football, to maintain dual careers as both athletes and professionals in other fields. This dual career path places additional demands on female athletes, as balancing employment with elite training can lead to greater burnout and emotional strain [88]. A survey of 726 female footballers from 12 countries and six confederations reported that approximately 71% of players were considered professional, 17% semi-professional with the remainder being amateur [89]. This survey also reported that 20% of players had caring responsibilities, including children, parents and partners, with many reporting caring for children when they themselves did not have children [89]. Staff working with female football players should be aware of both caring responsibilities and secondary employment outside of football and provide support and additional security where possible [89]. Social support systems, coping strategies and flexible work schedules can alleviate some of these burdens [90].

## Assessing Sleep: Screening and Monitoring

There are an increasing number of methods that may be used to assess sleep in athletes and include polysomnography, activity monitoring, wearable devices, sleep diaries and questionnaires. While the advantages and disadvantages of various objective and subjective measures have been outlined previously (for reviews, see [91, 92]), it is important to appreciate that the use of methods will likely vary depending on the goal of monitoring, the length of monitoring, specific characteristics of sleep assessed and resources available.

### Polysomnography

Polysomnography is commonly considered the gold standard for the assessment of sleep as it involves the determination of stages of sleep through measures of eye movement, brain electrical activity, heart rate, muscle activity, oxygen saturation, breathing rate and body movement [93]. Polysomnography is known to be the most accurate tool to assess rapid eye movement and non-rapid eye movement and is most often used to assess sleep disorders. While polysomnography has many advantages in the field of sleep medicine and in determining the effect of interventions to improve sleep, it is often limited in use because of laboratory access and the significant expense and expertise required for set-up and analysis.

### Wearable Devices

#### Traditional Research/Clinical Grade Actigraphy

Activity monitors determine sleep/wake based on recorded movement [94] and use specific algorithms for assessment [95]. Many of these devices have been validated for use in athletes [96, 97] and are practical for longitudinal monitoring. Given the practical nature of these devices, they have been commonly used in both research and practice. However, the standard research-grade activity monitoring devices do not allow for the assessment of sleep stages and do not include additional physiological measures. For this reason, activity monitoring in the field has become less popular and there has been a recent transition towards the use of wearable devices.

#### Consumer Grade Devices

Consumer grade devices typically include the assessment of sleep (sleep stages, sleep quality, sleep duration) as well as other overnight physiological measures (heart rate, heart rate variability, temperature). These devices most commonly connect to a smartphone application, which includes user-friendly interfaces and almost simultaneous analysis of data. For these reasons, consumer grade devices have become increasingly popular with both athletes and the wider population. However, it is important to appreciate the potential limitations of these devices, with limited accuracy, especially in terms of sleep staging, for many of the devices [98]. While some devices allow the restriction of data to the user, most devices provide large amounts of sleep and other physiological data to the user. This may result in orthosomnia, where the user becomes preoccupied and perfectionistic about their sleep, potentially increasing sleep disturbance. However, it is important to recognise that these devices and the associated algorithms (hardware and software) are rapidly progressing, and improvements are often observed when devices are upgraded and new models released.

### Sleep Diaries and Questionnaires

Given some of the challenges in using objective measures of sleep monitoring, there are advantages in using sleep diaries in terms of cost and simplicity. It is suggested that sleep diaries include measures of the following, for at least 7 nights: bed and wake time, lights out time, daytime napping, ratings of sleepiness and alertness, caffeine and alcohol intake, exercise and light-emitting device use [99]. While sleep diaries may be one of the more practical means of assessing sleep, biases may exist in terms of recall and truthfulness in responses [94]. For this reason, sleep diaries are often used in conjunction with objective sleep monitoring and can provide increased contextual information and accuracy when combined with objective measures. Sleep questionnaires are another simple means of assessing sleep and may be used as an initial sleep screen. Many commonly used sleep questionnaires were not designed for use in athletes and therefore, questionnaires designed specifically for athletes have been developed (Athlete Sleep Screening Questionnaire [100, 101] and Athlete Sleep Behaviour Questionnaire [102]). A more generic questionnaire to assess sleep quality, the PSQI, has been used in female soccer players, who were frequently classified as poor sleepers (PSQI score < 5) [82].

## Considerations for Sleep Monitoring

With the proliferation of sleep monitoring, in particular readily accessible consumer grade devices, there are increasing concerns regarding the large amount of data available to the athlete on a nightly basis. This may increase stress and anxiety associated with sleep and in effect increase sleep disturbance and subjective reports of poor sleep. It is important that practitioners working with athletes understand the limitations of commonly used sleep measuring tools and can provide education and feedback to athletes on the meaningfulness of the data. Wearable devices can contribute positively to awareness regarding sleep habits and may positively influence behavioural change; however, it is important that this is done in combination with professionals when required [103].

### Resource Availability

In addition to the importance of understanding current monitoring and assessment tools, it is also recognised that practitioners and staff may have varied access to resources for both assessing and improving sleep. Therefore, sleep monitoring and intervention tools may be stratified according to the availability of resources. The aim is to provide information to ensure that anyone wanting to assess and improve sleep, can do so appropriately regardless of the resources available (see Table 1).

**Table 1:** Methods to assess and improve sleep with consideration for low, medium and high resource availably

Resource availability	Sleep assessment
Low	Questionnaires (ASSQ, ASBQ, PSQI, sleep diary)
Medium	Wearables/activity monitors (without subscription)
High	Wearables (with subscription)
	Laboratory sleep assessment (PSG)
	In-home sleep assessment (partial PSG)
	**Sleep intervention**
Low	Online sleep education
Medium	Basic sleep education
High	Expert consultancy (sleep physician, scientist)
	Temperature-controlled mattresses
	Cognitive behavioural therapy for insomnia

*ASBQ* Athlete Sleep Behaviour Questionnaire, *ASSQ* Athlete Sleep Screening Questionnaire, *PSG* polysomnography, *PSQI* Pittsburgh Sleep Quality Index

## Sleep-Specific Recommendations for the Female Athlete

While there is currently limited research investigating sleep in female football players, available evidence suggests a number of recommendations for female athletes:

- Monitor and manage menstrual cycle symptoms and menstrual cycle dysfunction. Both a high number of menstrual cycle symptoms and menstrual cycle disorders have been linked to poor sleep.
- Understand potential effects of known negative influences on sleep, such as long-haul travel, caffeine, light exposure and late-night matches. Take into consideration mitigating interventions that may include consideration of flight schedules, scheduling of activities following a night match and judicious use of caffeine for performance.
- Determine an individual’s chronotype and preferred bed and wake times may be useful when considering room sharing.
- Consider monitoring sleep and potential interventions based on resource availability, time of season and player engagement.
- The minimum recommended duration for sleep monitoring is 7 days to ensure sleep data is captured on a weekend as well as weekdays. Monitoring sleep around extensive travel periods and congested schedules may also provide important information on sleep opportunities.

## Conclusions

While female-specific sleep research in football players is lacking, available evidence suggests that female individuals may have similar sleep to male individuals when measured objectively; however, they often report poorer sleep when measured subjectively. Menstrual cycle symptoms and menstrual cycle dysfunction may negatively influence objective and subjective assessments of sleep. Female football players and staff should be aware of the many potential external disturbances to sleep, such as travel, light exposure, caffeine, and caring responsibilities and consider various tools to measure and improve sleep with considerations for resource availability.
